# Development of a panel of serum IgG and IgA autoantibodies for early diagnosis of colon cancer

**DOI:** 10.7150/ijms.50169

**Published:** 2020-09-25

**Authors:** Meihong Chen, Xiaoqing Lin, Liangming Zhang, Lili Yu, Qingwei Wu, Songgao Zhang, Fangqin Xue, Yi Huang

**Affiliations:** 1 Provincial Clinical College, Fujian Medical University, Fuzhou 350001, China.; 2 Department of Clinical Laboratory, Fujian Provincial Hospital Jinshan Branch, Fuzhou 350001, China.; 3 Department of Clinical Laboratory, Fujian Provincial Hospital, Fuzhou 350001, China.; 4 Department of Gastrointestinal Surgery, Fujian Provincial Hospital, Fuzhou 350001, China.; 5 Center for Experimental Research in Clinical Medicine, Fujian Provincial Hospital, Fuzhou 350001, China.; 6 Key laboratory, Fujian Provincial Hospital, Fuzhou 350001, China.

**Keywords:** colon cancer, early diagnosis, autoantibody, combined detection

## Abstract

**Purpose:** Our pilot study in a small cohort by ELISA showed that the levels and positive rates of serum IgG autoantibodies against p53, HRAS and NSG1, and IgA autoantibody against TIF1γ in early colon cancer (CC) group were significantly higher than that of colon benign lesion (CBL) group / healthy control (HC) group (*P* <0.01), which suggested that four autoantibodies might be valuable for the diagnosis of patients with CC at early stage. On the basis of pilot study, we intend to comprehensively elucidate the performance of four autoantibodies for the early diagnosis of CC in a large sample cohort, and explore the optimal panel of autoantibodies in the diagnosis of patients with CC at early stage.

**Methods:** Western blot was used to define the ELISA results of serum anti-p53, HRAS, NSG1-IgG and anti-TIF1γ-IgA. The performances of anti-p53, HRAS, NSG1-IgG and anti-TIF1γ-IgA were evaluated by ELISA for the early diagnosis of CC with 601 serum samples of 157 patients with CC at early stage, 144 patients with CC at advanced stage, 130 patients with CBL, and 170 HC, and then the performances of different combinations of four autoantibodies were analyzed for the development of an optimal panel for the early diagnosis of CC.

**Results:** The results of anti-p53, HRAS, NSG1-IgG and anti-TIF1γ-IgA in western blotting were consistent with that in ELISA. The levels and positive rates of anti-p53, HRAS, NSG1-IgG and anti-TIF1γ-IgA in early CC group were significantly higher than that in CBL group/HC group (*P* <0.01), while had no significant difference from that in advanced CC group (*P* >0.05), of which anti-TIF1γ-IgA showed the highest area under the receiver operating characteristic curve (AUC) of 0.716 for the patients with CC at early stage, with 25.5% sensitivity and specificity at 96.7%. Additionally, a panel of anti-p53, HRAS-IgG and anti-TIF1γ-IgA showed the highest AUC among all possible combinations of four autoantibodies, up to 0.737, with 47.1% sensitivity at 92.0% specificity.

**Conclusions:** Serum IgG autoantibodies against p53, HRAS and NSG1, and IgA autoantibody against TIF1γ show the diagnostic value for the patients with CC at early stage, of which anti-TIF1γ-IgA is demonstrated to be a preferable biomarker, and an optimal panel comprised of anti-p53, HRAS-IgG and anti-TIF1γ-IgA might contribute to the further improvement of early diagnosis for CC.

## Introduction

Colon cancer (CC) is a common malignant tumor that seriously endangers human life and health. Globally, the incidence of colon cancer ranks 3^rd^ in males and 2^nd^ in females among all malignancies 1. Early diagnosis and timely surgical radical resection are of great significance for the prognosis of CC patients. Previous studies have shown that 5-year survival in patients with early CC who undergo surgery could reach as high as 97% 2. Unfortunately, due to the absence of apparent symptoms at the early stage, most of the patients present in the late stage of the disease, missing the best period for surgery.

As the traditional diagnostic method for colon cancer, fiber endoscopy and pathological tissue biopsies are applied in clinic for early CC detection, however, relatively complex operation, highly technical expertise requirement and uncomfortable invasive experience limit their application in the screening of asymptomatic populations. Therefore, improving the efficiency of early diagnosis of CC is becoming a clinical challenge 3. Serological biomarkers have been highly recommended in the screening for early CC due to non-invasive, convenient and safe advantages; nevertheless, current conventional tumor antigen markers (Carcinoembryonic Antigen (CEA), CA19-9 and CA724, etc.) meet a different degree of detection sensitivity or specificity problems, which limit their clinical value for diagnosing CC 4. Therefore, finding new serological markers for the early diagnosis of CC is essential.

Recent advances have demonstrated that autoantibodies against tumor-associated antigen (TAAs) exist in sera of 80% of cancer patients 3-5 years prior to the manifestation of clinical symptoms 5. Hence, autoantibody detection is expected to dramatically aid in the diagnosis of malignancies at early stage 5-6. Recently, using an approach based on HuProt array (v3.0; 20240 individual human proteins, provided by the High-throughput Biology Center of Johns Hopkins Medical College, USA), we successfully discovered and validated a total of eight IgG autoantibodies against p53, ETHE1, CTAG1A, C1QTNF1, TEX264, CLDN2, NSG1, and HRas, for lung cancer diagnosis at early stage 7. In addition, in agreement with the advances of serum IgA autoantibodies for the diagnosis of some malignancies 8-9, we also performed the screening of serum IgA autoantibody biomarkers by HuProt array and found some IgA autoantibodies with good diagnostic value for early lung cancer, of which IgA antibody against TIF1γ was demonstrated the optimal diagnostic performance for the patients with lung cancer at early stage by validation of ELISA assay 10.

In view of the apparent advantages of serum autoantibodies for the early diagnosis of malignancies, whether above 8 IgG autoantibodies and anti-TIF1γ IgA could also meet the diagnosis of early CC is worth exploring. Interestingly, by detecting a small cohort of serum samples comprised of 30 patients with early CC, 30 patients with benign colon lesions (CBL) and 30 healthy controls (HC) using ELISA assay, we found that levels of IgG antibodies against p53, HRAS, NSG1 and IgA antibody against TIF1γ, were significantly higher in patients with early CC than that in the CBL and HC group (*P* <0.01). This suggested that these four autoantibodies might be valuable for the diagnosis of patients with early CC. Based on our pilot observation, we intended to comprehensively evaluate the diagnostic performances of four autoantibodies in a large cohort of serum samples and then determine an optimal panel for the early diagnosis of CC by comparing the values of all possible combinations of four autoantibodies in this study.

## Material and methods

### Study subjects

157 patients with colon cancer (CC) at early stage (TNM 0/I/II stage), 144 patients with CC at advanced stage (TNM III/IV stage), 130 patients with colon benign lesion (CBL), and 170 healthy controls (HC) were recruited from February 2016 to December 2018 at Fujian Provincial Hospital. All patients strictly met the diagnostic standards recognized by international or professional societies and none of the patients had accepted any treatment for the malignancy; the clinical and pathological data were shown in **Table 1**. 170 HC participants received health examinations from the physical examination centre of Fujian Provincial Hospital and showed no evidence of disease, including malignancies, CBL, etc., based on the colonoscopy. All CBL and CC patients were confirmed by pathological examination of tissue biopsies under the colonoscopy. Each subject was collected 5 ml peripheral blood before the surgery and the serum was separated at 3000 rpm for 5 min and stored at -80 °C before use. This study was approved by the Institutional Review Board of Fujian Provincial Hospital, and all participants provided written informed consent.

### Detection of four TAAs by immunohistochemistry (IHC) staining

Formalin-fixed, paraffin-embedded 3-mm thick sections were deparaffinized and rehydrated, and then the sections were, respectively, incubated with primary antibodies against p53 (Abcam, UK) (1:100), HRAS (Abcam, UK) (1:100), NSG1 (Abcam, UK) (1:200), and TIF1γ (cell signaling technology, USA) (1:1000) for 18 h at 4 °C to carry out the standard IHC staining as described previously 10. The outcome of IHC staining for 30 pairs of CC tissues and matched paracancerous tissues, randomly selected from 157 cases with CC at early stage, was manually evaluated and scored by two independent certified pathologists. The intensity of staining was graded as: 0 = undetectable, 1+ = weak staining, 2+ = moderate staining and 3+ = strong staining.

### Identification of four serum autoantibodies by western blot

200 ng of denatured GST-tagged recombinant proteins, p53 (71 kDa), HRAS (46 kDa), NSG1 (48 kDa), and TIF1γ (150kD) at (expressed by yeast and provided by CDI Laboratories, Inc., USA) and 50 ng of denatured GST protein (26 kDa) were subjected to vertical electrophoresis on 12% SDS-PAGE at 20 mA, respectively. The separated proteins were electrotransferred onto the nitrocellulose membrane and incubated with sera (1:500 dilution for anti-p53, HRAS, NSG1-IgG detection, 1:100 dilution for anti-TIF1γ-IgA detection) or rabbit anti-GST antibody (Cwbiotech, China) (1:2000) at 4 °C overnight and the membranes were incubated with horseradish peroxidase-conjugated anti-human IgG (Cwbiotech, China) (1:5000), and anti-human IgA (Jackson Immuno, USA) (1:5000) to carry out the standard western blot assay as described previously 11. The immunoreactivity was visualized by chemiluminescence imager (BioRad, USA).

### Detection of four serum autoantibodies using ELISA assay

50 ng GST-tagged recombinant proteins, p53, HRAS, NSG1, and TIF1γ were coated onto 96-well plates at 4 °C overnight, respectively, and then the nonspecific binding was blocked by 3% BSA at 37 °C for 1 h. The wells were incubated with serum samples (1:500 dilution for anti-p53, HRAS, NSG1-IgG detection, 1:100 dilution for anti-TIF1γ-IgA detection) at 37 °C to carry out the standard ELISA assay as described previously 10, and immunoreactivity was measured by reading the A450. All assays were performed in duplicate, and the averaged OD value was calculated.

### Statistical analysis

SPSS 22.0 statistical software was used to analyze the experimental data. For individual autoantibodies, cut-offs based on mean+2SD of HC group were used and 95% binomial confidence intervals were calculated for positivity percentage (sensitivity). For a panel of autoantibodies, CC designation was made if each autoantibody level exceeded the mean+2 SD of HC group. Χ^2^ test was used for the comparison of rates between groups, and Mann-Whitney U-test was used for group comparisons. Graphpad Prism 5 and Medcalc v18.11 were used to draw charts and ROC curves, respectively. *P* <0.05 was considered a significant difference.

## Results

### Expressions of four TAAs in early CC tissues

**Figure 1:**
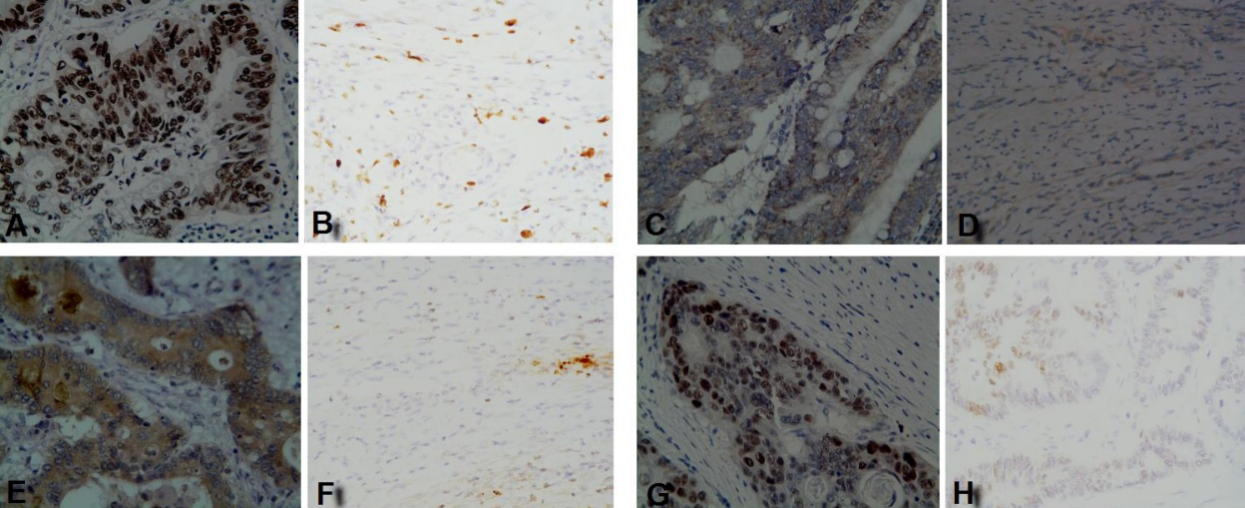
IHC staining results of four TAAs in early CC tissues and paracancerous tissues. **(A, C, E, G)** p53, HRAS, NSG1, and TIF1γ in early CC tissues, respectively; **(B, D, F, H)** p53, HRAS, NSG1, and TIF1γ in paracancerous tissues, respectively.

The IHC staining results showed that four TAAs were highly expressed in early CC tusses, of which p53 and TIF1γ proteins were expressed in cell nucleus, HRAS protein was expressed in both cytomembrane and cytoplasm, and NSG1 protein was expressed in cytoplasm, while all four TAAs proteins were negative or weakly expressed in matched paracancerous tissues (**Figure 1**). The IHC positive rates for 2+-3+ staining of p53, HRAS, NSG1, and TIF1γ in early CC tissues were 60.0%, 56.7%, 60.0% and 66.7%, respectively, and were significantly higher than 0.0%, 0.0%, 0.0% and 3.3% in paracancerous tissues (*P*<0.01, **Table 2**).

### Western blot validation of ELISA results

**Figure 2:**
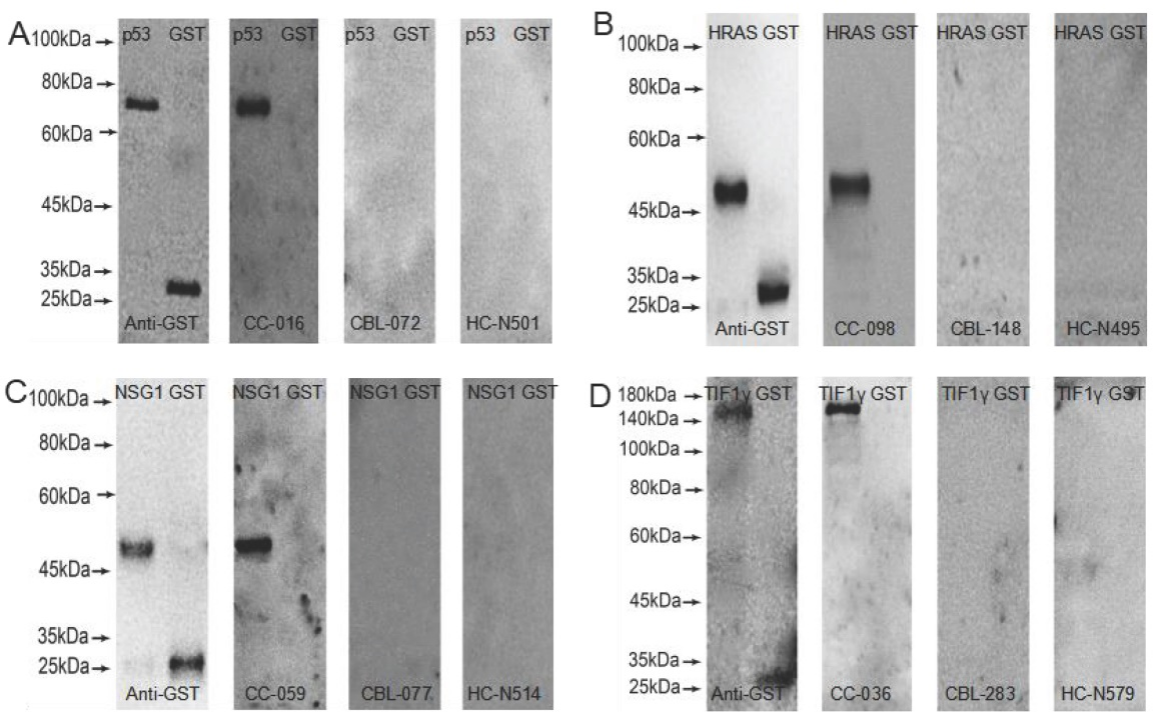
Western blot results of four serum autoantibodies. **(A)** p53-IgG; **(B)** HRAS-IgG;** (C)** NSG1-IgG; **(D)** TIF1γ-IgA.

GST-tagged recombinant proteins, p53, HRAS, NSG1, and TIF1γ were detected by western blot to validate the serum reactivity in ELISA, respectively. As shown in **Figure 2**, the serum of early CC patients with anti-p53, HRAS, NSG1-IgG (+), and anti-TIF1γ-IgA (+) detected by ELISA only bound to the target protein, and not to GST-tagged protein; the serum of CBL patients and HC with anti-p53, HRAS, NSG1-IgG (-), and anti-TIF1γ-IgA (-) detected by ELISA did not bind to the target protein or GST- tagged protein.

### Expressions of four serum autoantibodies

**Figure 3:**
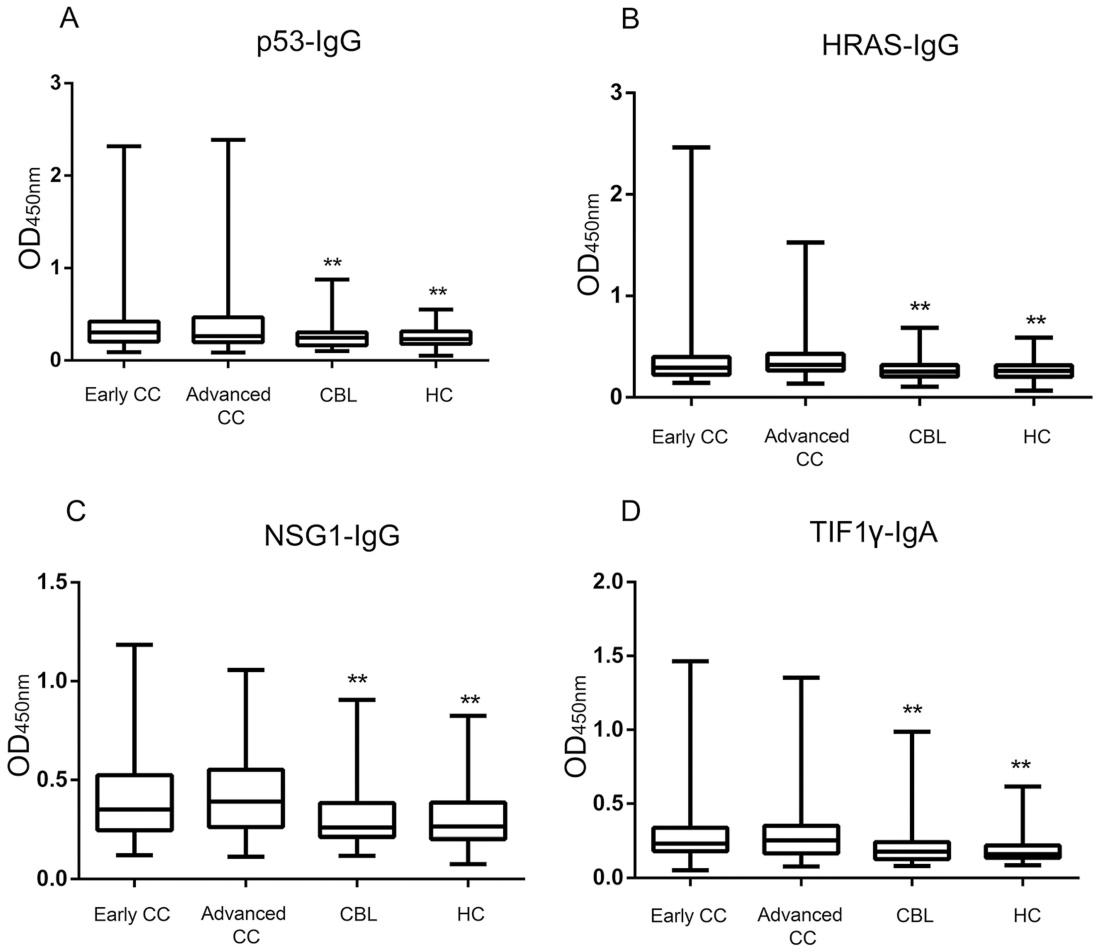
Comparison of four serum autoantibodies levels in early CC, advanced CC, CBL and HC groups. **: Compared with the early/advanced CC group, *P* <0.01.

**Figure 4:**
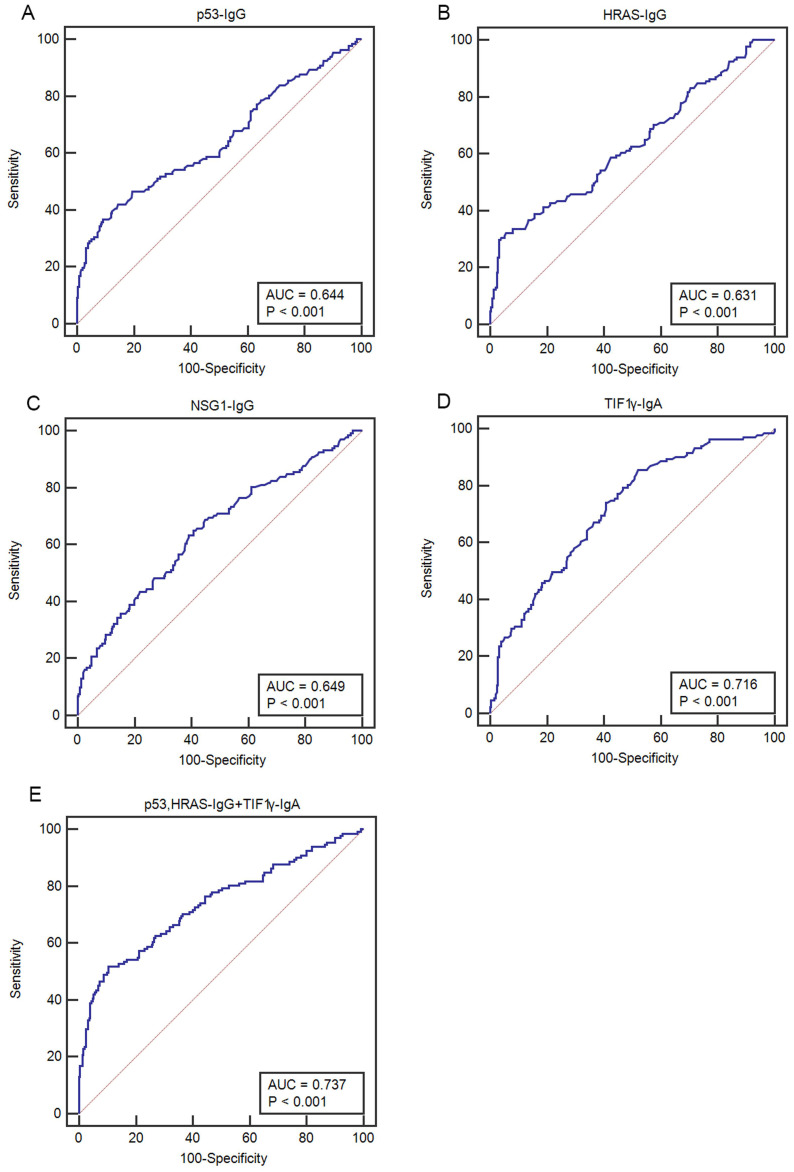
Comparison of ROC curves of four serum autoantibodies in patients with CC at early stage. **(A-D)** ROC curves for p53-IgG, HRAS-IgG, NSG1-IgG and TIF1γ-IgA, respectively; **(E)** ROC curve for an optimal panel of p53- IgG, HRAS-IgG and TIF1γ-IgA.

The ELISA results showed that the levels and positive rates of anti- p53, HRAS, NSG1-IgG and anti-TIF1γ-IgA in both early CC group and advanced CC group were significantly higher than that in CBL group and HC group (*P* <0.01), while there was no significant difference in the levels and positive rates of four autoantibodies between early CC group and advanced CC group (*P* >0.05) (**Figure 3, Table 3**). The AUC values of four autoantibodies for the patients with CC at early stage were >0.600, of which anti-TIF1γ-IgA showed the highest AUC of 0.716 (95%CI 0.668 - 0.761), with 25.5% sensitivity at 96.7% specificity (**Figure 4, Table 4**).

### Development of an optimal autoantibody panel

Considering the comprehensive evaluation of the performances among all possible combinations of four autoantibodies, a panel of anti-p53, HRAS-IgG and anti-TIF1γ-IgA showed the highest AUC for the patients with CC at early stage, up to 0.737 (95%CI 0.690-0.781), with 47.1% sensitivity at 92.0% specificity (**Table 4**).

## Discussion

An increasing number of evidences have represented that large amounts of autoantibodies could be released into sera due to the trigger of humoral immune responses for TAAs during early tumorigenesis. As compared with the TAAs in sera, serum autoantibodies against TAAs have demonstrated superior sensitivity for the early diagnosis of cancer in asymptomatic patients; additionally, TAAs-associated autoantibodies present high stability and persistence in sera, and are easy to detect 12. These excellent characteristics make it possible for TAAs-associated autoantibodies to serve as promising serological markers for the diagnosis of early CC 6. Especially mentioned, all TAAs related with four autoantibodies in this study are presented to be closely related with tumorigenesis. p53, a well-known tumor suppressor gene, is reported to be often mutated and overexpressed in various cancers, including CC 13-15, and responsible for the regulation of cell processes including cell cycle, apoptosis, senescence, and DNA repair 16-17. HRAS is a member of the ras oncogene family, whose mutations are involved in the occurrence of bladder, thyroid, salivary ductal, epithelial-myoepithelial, and kidney cancer 18-19. NSG1, an endosomal protein expressed in neuronal cells, is reported to be a transcriptional target of p53 based on the observation that some non-neuronogenous cancer cells can express NSG1 in a p53-dependent manner under the effect of hydrogen peroxide, doxorubicin, UV, and γ-ray. Furthermore, NSG1 overexpression presents the obvious effects on cell proliferation inhibition and cell apoptosis induction 20. TIF1γ, also known as TRIM33, ECTO, PTC7, and RFG7, is a regulatory factor of the TGF-β/Smad pathway by inhibiting Smad4 21-22. Recent existing studies have revealed that TIF1γ could play key role in the process of tumorigenesis by regulation of cell proliferation, differentiation, migration and invasion 23. By IHC staining, all four TAAs in this study are demonstrated to be highly expressed in tumor tissues of early CC patients at the protein level, which might contribute to the aberrant release of autoantibodies in sera of patients with CC at early stage. Of four TAAs-associated autoantibodies, anti-p53-IgG is reliably detectable in sera of patients with CC 24; however, the other three autoantibodies, anti-HRAS, NSG1-IgG and TIF1γ-IgA, have not yet been described in the CC diagnosis.

Considering our pilot observation that IgG autoantibodies against p53, HRAS and NSG1-IgG, and IgA autoantibody against IF1γ were highly expressed in sera of a small cohort of patients with early CC, we firstly proved the existence of a large amount of four autoantibodies in sera of early CC patients by the evidence that results of western blot were consistent with that of ELISA assay. And then, we performed the comprehensive evaluation of the diagnostic performances of four autoantibodies for patients with CC at early stage in a large cohort of up to 601 serum samples. Our results demonstrated the potentials of four autoantibodies for the early diagnosis of patients with CC. It was shown the levels and positive rates of four autoantibodies in early CC group were significantly higher than that in CBL and HC groups (*P* <0.01), of which anti-TIF1γ-IgA showed the best diagnostic performance of AUC of 0.716, with 25.5% sensitivity at 96.7% specificity for the patients with early CC. In addition, there were no significant difference in the levels and positive rates of four autoantibodies between early CC group and advanced CC group (*P* > 0.05), indicating the fact that autoantibodies might be preferable indicators of early diagnosis other than illness monitoring for CC.

In view of the sensitivity of individual detection of serum anti-p53, HRAS, NSG1-IgG, and anti-TIF1γ-IgA is relatively limited, we evaluated all possible combinations of these four autoantibodies. Encouragingly, the combination comprised of three autoantibodies, anti-p53, HRAS-IgG, and anti-TIF1γ-IgA, was determined to be an optimal panel for the diagnosis of early CC, supported by its potential of improving AUC to 0.737, with up to 47.1% sensitivity at 92.0% specificity for early CC. Finally, to the best of our knowledge, the diagnostic value of this panel of anti-p53, HRAS-IgG, and anti-TIF1γ-IgA for early CC should be further proven by a multi-center research in the future and the further studies would be expected to carry out to excavate more valuable IgG and IgA autoantibodies to enhance the sensitivity and specificity for the diagnosis of early CC, so as to further improve the early diagnostic efficiency and 5-year survival rate of CC.

## Figures and Tables

**Table 1:** Clinical data for the early CC, CBL and HC groups

	CC (n = 301)	CBL (n = 130)	HC (n = 170)	*P*
Age (years)	62.1±7.2	62.1±8.3	61.0±7.7	0.162
**Sex (%)**				0.551
Male	188 (62.3)	83 (63.5)	111 (65.1)	
Female	113 (37.7)	47 (36.5)	59 (34.9)	
**Staging (%)**				
0	24 (8.0)			
I	36 (12.0)			
II	97 (32.2)			
III	107 (35.5)			
IV	37 (12.3)			
CB**L (%)**				
Colonic adenoma		56 (43.1)		
Colon hyperplastic polyps		72 (55.4)		
Colon tuberculosis		2 (1.5)		

**Table 2:** Comparison of positive rates of four TAAs expressions between early CC tissues and paracancerous tissues

	n	Early CC tissues		Paracancerous tissues	
		2+-3+	0-1+	2+-3+	0-1+
p53	30	60.0%(18/30)^**^	40.0%(12/30)^**^	0.0%(0/30)	100%(30/30)
HRAS	30	56.7%(17/30)^**^	43.3%(13/30)^**^	0.0%(0/30)	100%(30/30)
NSG1	30	60.0%(18/30)^**^	40.0%(12/30)^**^	0.0%(0/30)	100%(29/30)
TIF1γ	30	66.7%(20/30)^**^	33.3%(10/30)^**^	3.3%(1/30)	96.7%(29/30)

**Notes:** **indicates compared with Paracancerous tissues, *P* <0.01.

**Table 3:** Comparison of the positive rates of four serum autoantibodies in early CC, advanced CC, CBL and HC groups

Marker	Early CC (n = 157)	Advanced CC (n = 144)	CBL (n = 130)	HC (n = 170)
	Positive rate (%)	Positive rate (%)	Positive rate (%)	Positive rate (%)
p53-IgG	23.6(37/157)^**^	27.8(40/144)^**^	4.6(6/130)	2.4(4/170)
HRAS-IgG	18.5(29/157)^**^	20.8(30/144)^**^	3.8(5/130)	2.4(4/170)
NSG1-IgG	20.4(32/157)^**^	22.2(32/144)^**^	6.2(8/130)	3.5(6/170)
TIF1γ-IgA	25.5(40/157)^**^	25.7(37/144)^**^	4.6(6/130)	2.4(4/170)

**Notes:** **indicates compared with CBL group/HC group, *P* <0.01.

**Table 4:** Comparison of the performances of four serum autoantibodies in diagnosing the patients with CC at early stage

Marker	Sensitivity (%)	Specificity (%)	AUC	SE	95% CI	*P*
p53-IgG	23.6	96.7	0.644	0.032	0.594-0.692	<0.001
HRAS-IgG	18.5	97.0	0.631	0.031	0.580-0.679	<0.001
NSG1-IgG	20.4	95.3	0.649	0.030	0.599-0.697	<0.001
TIF1γ-IgA	25.5	96.7	0.716	0.027	0.668-0.761	<0.001
p53, HRAS-IgG	34.4	94.0	0.680	0.031	0.630-0.726	<0.001
p53, NSG1-IgG	38.2	92.0	0.688	0.031	0.639-0.734	<0.001
p53-IgG +TIF1γ-IgA	40.8	93.7	0.728	0.029	0.680-0.772	<0.001
HRAS, NSG1-IgG	30.6	93.0	0.664	0.031	0.614-0.711	<0.001
HRAS-IgG+TIF1γ-IgA	35.7	95.3	0.724	0.028	0.676-0.769	<0.001
NSG1-IgG+TIF1γ-IgA	35.7	92.7	0.710	0.028	0.662-0.755	<0.0001
p53, HRAS, NSG1-IgG	44.6	90.0	0.695	0.031	0.646-0.741	<0.001
p53, HRAS-IgG+TIF1γ-IgA	47.1	92.0	0.737**	0.029	0.690-0.781	<0.001
p53, NSG1-IgG+TIF1γ-IgA	46.5	89.7	0.727	0.029	0.680-0.771	<0.001
HRAS, NSG1-IgG+TIF1γ-IgA	42.0	91.3	0.722	0.029	0.675-0.767	<0.001
p53, HRAS, NSG1-IgG+TIF1γ-IgA	51.6	88.0	0.735	0.029	0.688-0.779	<0.001

**Notes:** ** indicate the highest AUC among all possible combinations of four autoantibodies; **Abbreviations:** AUC, area under the curve; SE, standar error; CI, confidence interval.
